# Habitat Integrity Challenges for the Chinese Alligator Amid Land Occupation by Human: Pathways for Protection

**DOI:** 10.1002/ece3.71113

**Published:** 2025-03-10

**Authors:** Ke Sun, Meng Li, Ziyi Wang, Siqing Sun, Jiayue Yang, Xiaobing Wu, Tao Pan

**Affiliations:** ^1^ College of Life Sciences Anhui Normal University Wuhu China; ^2^ Anhui Chinese Alligator National Nature Reserve Xuancheng Anhui China; ^3^ National Long‐Term Scientific Research Base for Chinese Alligator Artificial Breeding and Protection in Anhui Anhui Research Center for Chinese Alligator Reproduction Xuancheng China

**Keywords:** Chinese alligator, ecological corridor, habitat integrity, landscape, restoration suggestion, suitability

## Abstract

Effective conservation of endangered species necessitates not only the preservation of core habitats but also the enhancement of landscape connectivity. As a critically endangered Crocodylia, the Chinese alligator ( *Alligator sinensis* ) strongly relies on the fragmented wetland habitat of the lower area of the Yangtze River. The integrity of its habitat needs evaluating, and the connectivity restoring plan needs designing. In this study, we estimated the suitability of the habitat in the lower area of the Yangtze River using a Maxent model. Then, the potential ecological corridors between each nature reserve were selected by the least‐cost path and circuit theory methods, and the landscape connectivity was analyzed. The results showed that the highly suitable habitat had a low integrity and was fragmented into small pieces by residential areas, farmland, and mountain areas. Four priority ecological corridors (i.e., Xiadu‐Hongxing, Changle‐Zhongqiao, Zhongqiao‐Shuangkeng, and Hongxing‐Shuangkeng) were selected. The land occupation of humans seriously impacts the integrity of the Chinese alligator, and the unsuitable forest and artificial landscapes along the corridors indicate the need for a massive habitat restoration project. The landscape connectivity of the habitat needs to be progressively restored to provide more possibilities for the dispersal of the Chinese alligator.

## Graphical abstract

The integrity of the Chinese alligator habitat was low. The man‐made landscape decreased the suitability of the habitat and limited the disposal of the Chinese alligator. Constructing the ecological corridors was difficult due to human activity, long distance, and slow diffusion speed of the alligator.

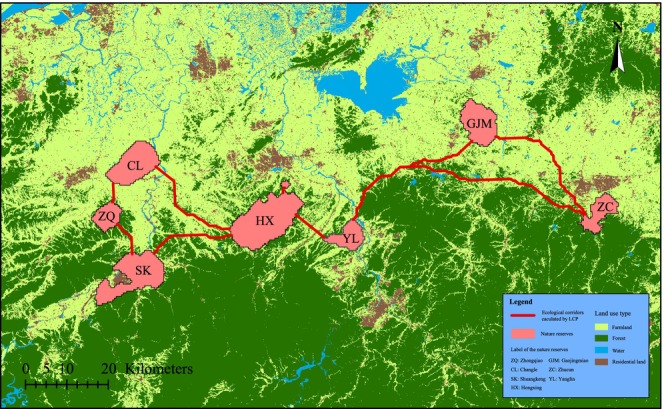

## 1 Introduction

Habitat integrity plays a vital role in the survival, gene exchange, and extinction risk of endangered animals (Li et al. 2024). Wetlands, key habitats for reptiles (Roe and Georges 2007), are being replaced by artificial landscapes due to rapid economic and social development (Salviano et al. 2021), which results in serious challenges to the habitat integrity of endangered animals. As an endangered large carnivorous reptile, the Chinese alligator has high requirements for habitat and food. Although the conservation work is well regarded by the Chinese government, human occupation of the native habitat and concerns about the safety of life and property still lead to a conflict between the population spread of the Chinese alligator and human interests.

The Chinese alligator ( *Alligator sinensis* ) is classified as critically endangered by the International Union for Conservation of Nature's red list of threatened species, with only about 1000 wild individuals left (Pan et al. 2019). The habitat of the Chinese alligator is distributed in one of the important economic zones—the lower reaches of the Yangtze River, and the rapid urbanization process in this zone has led to serious habitat fragmentation of the Chinese alligator. High‐integrity habitat helps crocodiles form stable populations, such as the American alligator and caimans (Eversole and Henke 2022). Among the Crocodilia, the distribution area of the Chinese alligator should be given the highest priority for protection (Lourento‐de‐Moraes et al. 2023).

In order to protect this endangered species, nature reserves have been established to provide key habitats for this species. The habitat of the Chinese alligator is dominated by wetlands, and the landscape of the current nature reserves is mostly agricultural ponds and lakes with artificial facilities (Maqsood and Rong 2019). However, most of these reserves have been isolated for a long time due to regional development and management limitations, reducing genetic exchange among the populations (Morandi et al. 2020; Merenlender et al. 2022). Moreover, habitat fragmentation, artificial landscape barriers, and human fear of alligators could be obstacles to the dispersal of the Chinese alligator. The design and establishment of ecological corridors play an important role in decreasing the barriers to species dispersal (Baguette et al. 2013). By analyzing the feasibility of ecological corridors, priority corridors can be defined, which can provide a reference for environmental planning and policy (Salviano et al. 2021). However, there is still a lack of habitat integrity evaluation and a feasible plan for ecological corridor construction.

To evaluate the habitat integrity of the Chinese alligator and provide actionable conservation planning, this study assessed the integrity of the habitat under the influence of land occupation by humans and analyzed the options for ecological corridor construction. An ecological niche modeling approach using MaxEnt was applied to identify the potential distributions of suitable habitats and dispersal resistance. Then, ecological corridors for isolated Chinese alligator reserves were identified using the weighted cost distance and the circuit theory method, and the landscape characteristics of the priority ecological corridors were analyzed.

## 2 Methods

### 2.1 Study Area

**FIGURE 1:**
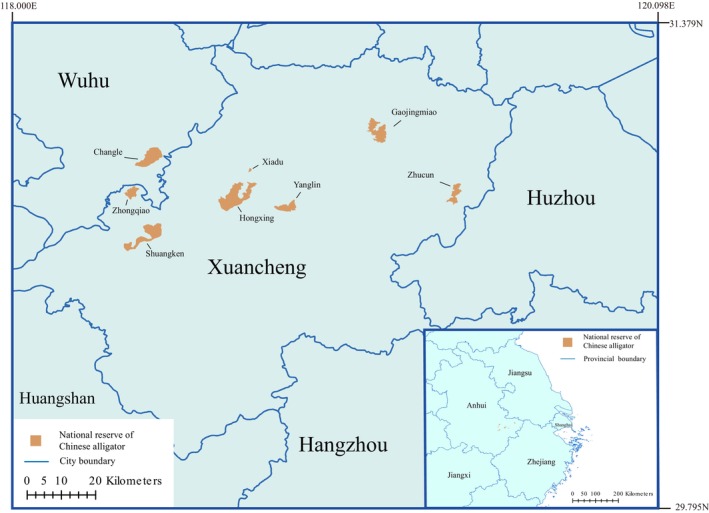
Distribution of nature reserves of Chinese alligator.

The economic zone of the lower reaches of the Yangtze River consists of Jiangsu Province, Zhejiang Province, Anhui Province, and Shanghai City, and it is the historical distribution area for Chinese alligators. However, due to economic development in this region, Chinese alligators are now only distributed in some parts of Anhui and Zhejiang provinces. To fully assess the impact of climate change and land occupation by humans on the distribution of Chinese alligators, Anhui Province, Jiangsu Province, Zhejiang Province, and Shanghai City in China were selected as the study area (Figure 1). The latitude and longitude range of the study area is 27.1583°–35.1333°N, 114.8750°–122.5250°E, and the total area is 343805.28 km^2^. There are a total of eight Chinese alligator core reserves in Xuancheng City and Wuhu City, Anhui Province, namely Zhongqiao, Shuangkeng, Xiadu, Hongxing, Yanglin, Gaojingmiao, Zhucun, and Changle. These eight reserves cover an area of 196.45 km^2^, with the Yangtze River to the south and the Huangshan Mountains to the north.

### 2.2 Distribution of the Chinese Alligator

The alligator distribution data was obtained from a survey of wild Chinese alligators in May 2022 organized by the Administrative Bureau of Anhui Chinese Alligator National Nature Reserve, and used in this study with permission. The survey area included eight natural reserves. In this survey, Chinese alligators in each lake were investigated using the spotlight survey method, and the locations of observed Chinese alligators were recorded. A total of 353 alligator locations were obtained in this study.

### 2.3 Habitat Suitability of the Chinese Alligator

In order to evaluate the habitat suitability, appropriate environmental variables need to be selected. Factors of climate, landscape, and human activities are regarded as the major factors influencing the distribution of alligators (Beal and Rosenblatt 2020; Balaguera‐Reina et al. 2024). Therefore, environmental variables reflecting these three factors are selected for subsequent analysis.

**FIGURE 2:**
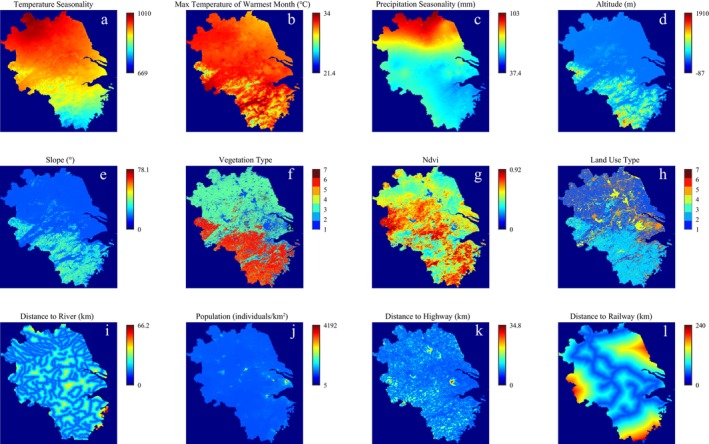
Input data of environmental variables. (a) Temperature seasonality; (b) maximum temperature of the warmest month; (c) precipitation seasonality; (d) altitude; (e) slope; (f) vegetation type; (g) NDVI; (h) land use type; (i) distance to river; (j) population; (k) distance to highway; (l) distance to railway.

Among the climate variables, temperature and precipitation have significant effects on the distribution and behavior of the Chinese alligator (Tu et al. 2023; Yang et al. 2024). Considering the correlation between the climate variables (Results of multiple correlation analysis are shown in Figure S1) and the influencing mechanisms, temperature seasonality, the maximum temperature of the warmest month, and precipitation seasonality were selected as environmental variables of climate. Among the variables of landscape and human activities, slope, vegetation coverage, and distance to water area can influence nest‐site use of the Chinese alligator (Wang et al. 2011), and anthropogenic structures can reduce the distribution of alligators (Rosenblatt et al. 2023). Therefore, slope, altitude, vegetation type, Normalized Difference Vegetation Index (NDVI), land use type, and distance to river, population, distance to highway, and distance to railway were also selected as environmental variables. Then, these 12 environmental variables (Table 1) were used to establish a MaxEnt model. The distribution of each environmental variable in the study area is shown in Figure 2. ArcGIS Pro 2.7 software (Esri, USA) was used to clip the raster layer of each environmental variable layer to the study area, with a resolution of 2086 × 2000 pixels. The locations of the Chinese alligator were obtained from a comprehensive survey, and the data were not spatially offset. All 353 Chinese alligator locations and 12 environmental variable layers were used to establish the MaxEnt model using MaxEnt 3.4.4 in the Java platform (https://biodiversityinformatics.amnh.org/open_source/maxent). The maximum number of iterations was set to 2000, the background points were set to 10,000, the random test percentage was set to 25%, the replicated run type was set to ‘Cross validate’, the number of replicates was set to 4, the output format was ‘Cloglog’, and the output file type was ‘asc’. The accuracy of the model was verified by the area under the curve (AUC) of the receiver operating characteristic curve (Van Van et al. 2011). A higher AUC value indicates higher predictability of the model, and the prediction accuracy is high when the AUC value is > 0.9 (Liu et al. 2024). The output distribution map was reclassified into four levels: unsuitable area (0–0.1), poorly suitable area (0.1–0.31), moderately suitable area (0.31–0.6), and highly suitable area (0.6–1) (Ji et al. 2020). After the model was established, the complementary log–log response curves of the environmental variables were recorded.

**TABLE 1:** Influence factors of the disposal of Chinese alligator.

Category	Factors	Data range	Resource	Year
Climate	Temperature seasonality	669–1010	World Meteorological Database http://worldclim.org/	2020
	Max temperature of warmest month	21.4°C–34°C	World Meteorological Database http://worldclim.org/	2020
	Precipitation Seasonality	37.4–103 mm	World Meteorological Database http://worldclim.org/	2020
Terrain	Altitude	−87–1910 m	DEM (resolution: 90 × 90 m) http://www.webmap.cn/	2008
	Slope	0°–78.1°	Calculated using Altitude	
Landscape	Vegetation type	(1) Bare land; (2) Grass; (3) Farmland; (4) Shrub; (5) Coniferous Forest; (6) Broad‐leaved Forest; (7) Wetland	GLC_FCS30 (Zhang, et al., 2021)	2022
	NDVI	0–0.92	2000–2020 China 30 m annual maximum NDVI dataset (Yang, et al., 2019)	2022
	Land use type	(1) Farmland; (2) Forest; (3) Grass; (4) Water; (5) Residential land; (6) No used	China land use and land cover change database http://www.resdc.cn	2022
	Distance to river	0–66.2 km	Calculated with Land use type using Arcgis Pro	
Anthropic	Population	5–4192 individuals/km^2^	Population density data sets for Chinese provinces www.worldpop.org	2020
	Distance to highway	0–34.8 km	Calculated with Land use type using Arcgis Pro	
	Distance to railway	0–240 km	Calculated with Land use type using Arcgis Pro	

### 2.4 Calculation of the Dispersal Resistance Distribution

The weighting coefficient of the different environmental variables used for the dispersal resistance distribution calculation was usually provided by experts in this study area (Lamounier et al. 2024) or calculated based on the species distribution sites. Among the two methods, weighting coefficients determined by the actual species distribution sites are more accurate (Naidoo et al. 2018). In this study, the resistance layers and their weighting coefficients for dispersal resistance distribution calculation were determined according to the importance value of environmental variables to the MaxEnt model, which was built using the actual species distribution sites. The calculating method was as follows.

1. Based on the results of the MaxEnt model, the environmental variables with higher importance were selected as resistance factors, and the resistance layers and weighting coefficients were calculated. However, residential areas, highways, and railways can seriously hinder animal migration (Serieys et al., 2021, Zhuo et al., 2022); these factors need to be weighted separately.
2. Based on the opinions of the Chinese alligator expert group in Anhui Province, which has 30 years of research experience on Chinese alligators, the weighting coefficients of the land use type, the road distance, and the railway distance were set to 0.1, 0.1, and 0.2, respectively. The total weighting coefficient of other resistance values was set to 0.6. The resistance layer of each environmental variable was calculated using the following formula.

(1) $$\mathrm{RL}\left(x,y\right)=1-\text{Resample}\left(\mathrm{EV}\left(x,y\right)\right)$$

Where *RL*(*x*, *y*) is the resistance value on the coordinates (*x*, *y*) of resistance layer; *EV* (*x*, *y*) is the environmental value on the coordinates (*x*, *y*) of environmental value layer; *Resample*(*EV*(*x*, *y*)) is the result of resampling on the complementary log–log response curve using $\mathrm{EV}\left(x,y\right)$.

(3) The layer of dispersal resistance distribution was calculated using the following formula.

(2) $$\begin{array}{c} \mathrm{DRD}\left(x,y\right)=\sum_{i=1}^{n}\frac{0.6\times {\mathrm{Imp}}_{i}}{\sum_{i=1}^{n}{\mathrm{Imp}}_{i}}\mathrm{RL}{\left(x,y\right)}_{i}+0.1\times \mathrm{RL}{\left(x,y\right)}_{\mathrm{LUT}}\\ +0.1\times \mathrm{RL}{\left(x,y\right)}_{\mathrm{RDD}}+0.2\times \mathrm{RL}{\left(x,y\right)}_{\mathrm{RLD}} \end{array}$$

Where *DRD*(*x*, *y*) is the resistance value at the coordinates (*x*, *y*) of the layer of dispersal resistance distribution; *i* is the number of selected resistance factors; *Imp* is the importance value of the environmental variable; *RL*(*x*, *y*)_LUT_ is the *RL*(*x*, *y*) of land use type; *RL*(*x*, *y*)_RDD_ is the *RL*(*x*, *y*) of road distance; and *RL*(*x*, *y*)_RLD_ is the *RL*(*x*, *y*) of railway distance. Finally, this dispersal resistance distribution map was normalized by the Max‐min method.

### 2.5 Prediction and Analysis of the Ecological Corridors

The determination of the core areas is critical for the prediction of ecological corridors, and core areas can be obtained by segmenting the heat map by suitability, which is an output of the MaxEnt model (Chibeya et al. 2021; Shrestha et al. 2021). However, the Chinese alligator reserves were relatively isolated. The main purpose of establishing an ecological corridor is to connect the reserves; thus, eight reserves were used as the core areas to calculate the ecological corridors.

Considering the dispersal ability, it is generally necessary to set a buffer zone (Yang et al. 2023; McCluskey et al. 2024) at a certain distance from the periphery of the core area. Based on the non‐uniformity of the dispersal resistance that was due to the dispersal ability of the Chinese alligator and the human population density, the core areas were expanded to twice the original area using the flood fill method.

The least‐cost path (LCP) based on path‐through analysis and circuit theory based on random walk theory are common methods for designing ecological corridors (Marrotte and Bowman 2017; Dickson et al. 2019). The LCP method uses a search kernel to find the pathway with the lowest accumulated cost of resistance raster (Hoover et al. 2020), whereas the circuit theory method can identify movement paths using current flowing between nodes to simulate the movement of an individual (Merrick and Koprowski 2017). These two methods are suitable for species with high sensitivity to the climate and landscape (Diniz et al. 2020). In this study, ArcGIS Pro 2.7 software and Linkage mapper toolbox 3.0 (https://linkagemapper.org) were used to predict the potential ecological corridors between the core areas using the LCP and current theory methods, respectively (Iannella et al. 2024). The dispersal resistance distribution map from section 2.4 was used as the resistance surface, the weighted cost distance of corridor width was set as 2 km, and the weighted cost distance threshold for separating the ecological corridors was 20 km. Then, ArcGIS Pro 2.7 software was used to expand the corridor line that was obtained by the LCP method, with a width of 2 km set as the corridor range of the LCP method. The current density map that was obtained by the current theory method was segmented with a threshold of 0.02 as the corridor range of the circuit theory method. Finally, the area of the land use types in each corridor was counted and compared.

## 3 Results

### 3.1 Results of MaxEnt Model

**FIGURE 3:**
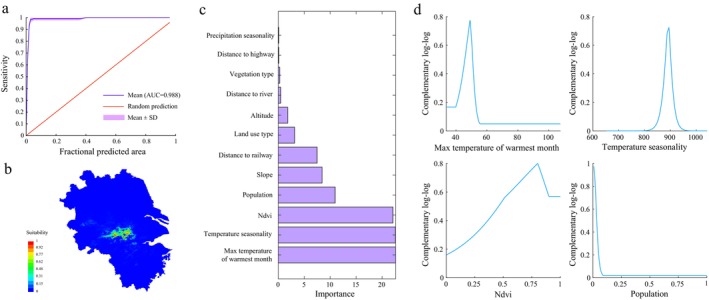
Results of maxent model for suitability evaluation of Chinese alligator. (a) Sensitivity (1—omission); (b) suitability evaluating result; (c) importance of all input environmental variables; (d) complementary log–log of the four most important input environmental variables.

As shown in Figure 3a,b, the average test AUC of cross‐validation of the MaxEnt model was 0.988, and the highly suitable areas were concentrated in the southern part of Anhui Province. The importance value of each variable to the MaxEnt model establishment is shown in Figure 3c. The importance of the maximum temperature of the warmest month, temperature seasonality, NDVI, and population to the model was relatively high, and the variation in the complementary log–log of these four environmental variables is shown in Figure 3d. The optimal maximum temperature of the warmest month was 49.0°C, the optimal temperature seasonality was 894, the optimal NDVI was 0.8, and the optimal population was 0. Therefore, the maximum temperature of the warmest month, temperature seasonality, NDVI, and population were selected as resistance factors, and the weighting coefficients of these factors were 0.174, 0.173, 0.169, and 0.084, respectively.

### 3.2 Integrity of the Chinese Alligator Habitat

**FIGURE 4:**
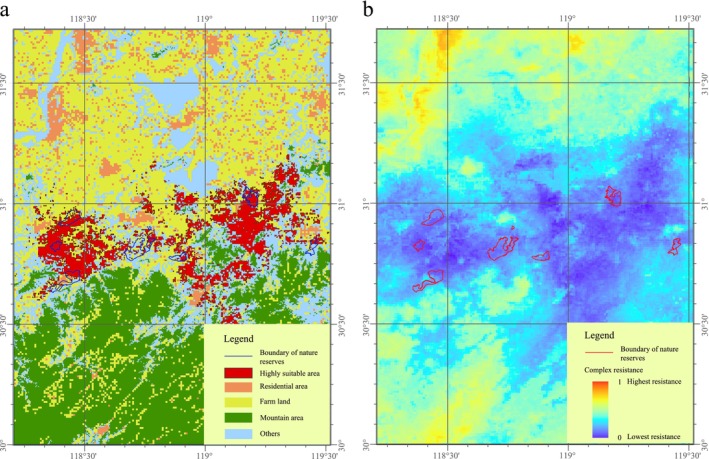
Effects of human use on the suitability of Chinese alligator and the compound resistance map. (a) Distribution of highly suitable area and human use area; (b) compound resistance map.

The highly suitable habitat distribution in the study area that was predicted by the MaxEnt model is shown in Figure 4a. The area of highly suitable habitat was 1582.3 km^2^, and it was mainly located in the lower reaches of the Yangtze River in Anhui Province. As shown in Figure 4a, the highly suitable habitat had low integrity and was fragmented into small pieces by residential areas, farmland, and mountain areas. Even in some of the nature reserves, such as Shuangkeng, Hongxing, Yanglin, and Zhucun, the highly suitable habitat made up less than half of the proportion of the total habitat. The distribution of the Chinese alligator dispersal resistance is shown in Figure 4b. The low resistance area was almost the same area as that of the highly suitable habitat for the Chinese alligator, and the residential areas, farmland, and mountain areas increased the dispersal resistance in the nearby areas. After the influence of terrain, land occupation by humans was the second most important factor in decreasing the suitability of the habitat and limiting the dispersal of the Chinese alligator.

### 3.3 Potential Ecological Corridors

**FIGURE 5:**
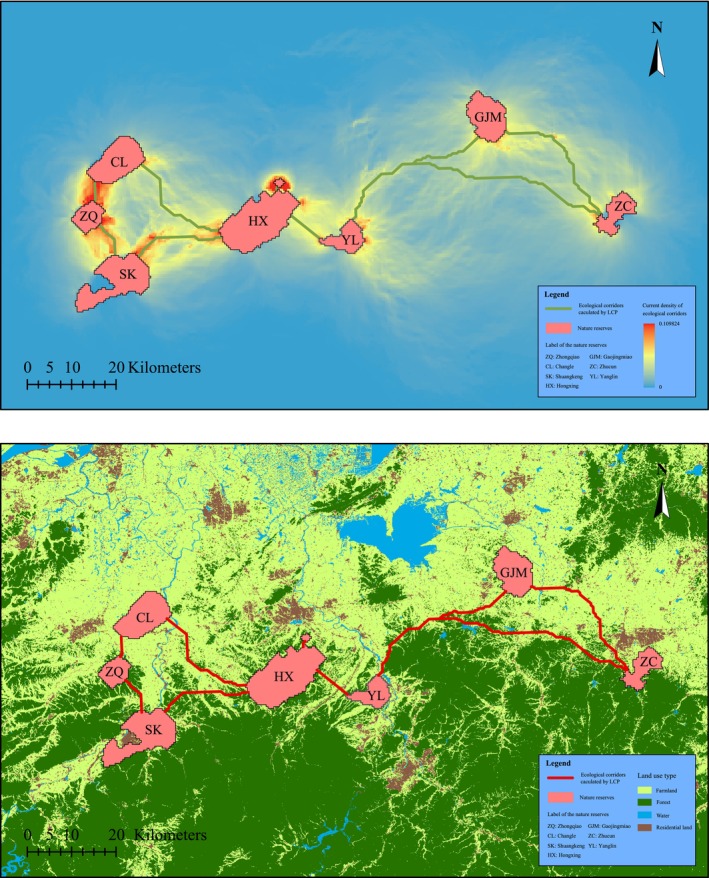
Ecological corridors between nature reserves of the Chinese alligator. (a) Ecological corridors calculated using the LCP method; (b) ecological corridors calculated using current theory.

The potential ecological corridors between these eight reserves are shown in Figure 5. The thermal map in Figure 5a represents the ecological corridor predicted by the circuit theory method, while the red line in Figure 5b represents the ecological corridor predicted by the LCP method. A total of nine ecological corridors were predicted, and the range of these ecological corridors avoided densely populated areas and mountainous areas. The ecological corridors in the east, which connected the Changle, Zhongqiao, Shuangkeng, Hongxing, and Xiadu reserves, were relatively short, while the ecological corridors connected to Gaojingmiao and Zhucun were relatively long and would be more difficult to create. The parameters of the nine ecological corridors are shown in Table 2. The four ecological corridors with the lowest cost‐weighted distance (CWD) were Xiadu‐Hongxing, Changle‐Zhongqiao, Zhongqiao‐Shuangkeng, and Hongxing‐Shuangkeng, and their CWDs were 213.5, 498.9, 646.0, and 1171.8 m, respectively. The CWD of the corridor between Hongxing and Yanglin was also relatively low, but the current density was not as high as that of the four corridors above, as shown in Figure 5a. According to the results of the LCP and current theory methods, the ecological corridors between Xiadu‐Hongxing, Changle‐Zhongqiao, Zhongqiao‐Shuangkeng, and Hongxing‐Shuangkeng were selected as the priority ecological corridors.

**TABLE 2:** Parameters of potential ecology corridors.

ID	Name	Euclidean distance / (m)	Cost‐weighted distance/(m)	Length of least‐cost path /(m)
1	Xiadu‐ Hongxing	743	213.5	1397
2	Changle‐Zhongqiao	4117	498.9	4672
3	Zhongqiao‐Shuangkeng	6695	646.0	8556
4	Hongxing‐Shuangkeng	15,682	1171.8	18,819
5	Hongxing‐Yanglin	6943	1427.2	8105
6	Chagnle‐Hongxing	20,681	2098.3	24,539
7	Gaojingmiao‐Yanglin	30,546	2945.6	35,580
8	Gaojingmiao‐Zhucun	25,639	3090.8	31,259
9	Zhucun‐Shuangkeng	44,736	5635.0	61,050

### 3.4 Landscape Characteristics of the Priority Ecological Corridors

**FIGURE 6:**
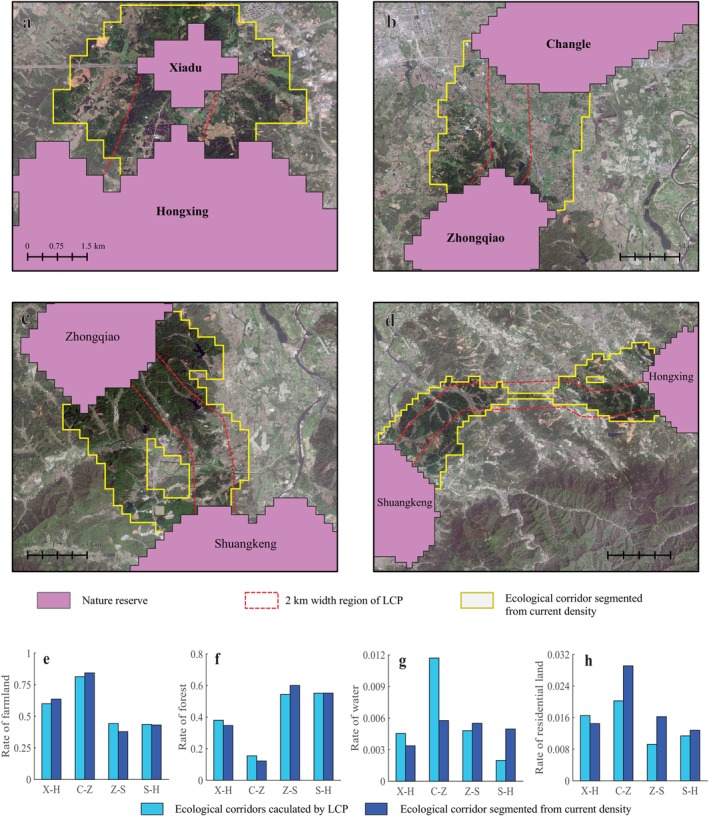
Landscape of priority ecological corridors. (a) Ecological corridor between Xiadu and Hongxing; (b) ecological corridor between Changle and Zhongqiao; (c) ecological corridor between Zhongqiao and Shuangkeng; (d) Ecological corridor between Shuangkeng and Hongxing; (e) rate of farmland of priority ecological corridors; (f) rate of forest of priority ecological corridors; (g) rate of water of priority ecological corridors; (h) rate of residential land of priority ecological corridors; X‐H: Xiadu‐Hongxing; C‐Z: Changle‐Zhongqiao; Z‐S: Zhongqiao‐Shuangkeng; S‐H: Shuangkeng‐Hongxing.

The landscape of these priority ecological corridors is shown in Figure 6a–d, and the proportion of farmland, forest, water, and residential land in the range of the priority corridor landscape is shown in Figure 6e–h. The ecological corridor between Xiadu and Hongxing was short, the proportion of farmland was relatively high, and there were many ponds; thus, it would be relatively easy to build this ecological corridor. The ecological corridor between Changle and Zhongqiao had the highest proportion of farmland, water, and residential land, and the proportion of mountain area and forest was relatively small. This ecological corridor is suitable for corridor reconstruction, but the relationship between Chinese alligator protection and agricultural activity should be considered. The ecological corridor between Zhongqiao and Shuangkeng had a high proportion of mountain area and forest, so it will be necessary to design and develop ponds in the mountain forest for ecological corridor reconstruction. While the ecological corridor between Shuangkeng and Hongxing was relatively long, the proportion of forest in the mountainous areas was relatively high, and there was an ecological pinch point in the middle of this corridor, which may be caused by the high elevation. This would make it difficult for this ecological corridor to be built.

## 4 Discussion

Improving the integrity and connection of habitats is crucial for the protection of endangered animals (Santiago et al. 2018). However, according to the results that were observed in this study, the habitat of the Chinese alligator is currently limited to parts of the lower reaches of the Yangtze River and is separated by residential areas and farmland. Moreover, the proportion of mountains and artificial landscape in the ecological corridors between the nature reserves is relatively high, which greatly limits the spread of the Chinese alligator between the reserves. Therefore, decreasing human activity intensity and improving the micro‐landscape (Bai et al. 2020) in these nature reserves are urgently needed to enhance the integrity of the Chinese alligator habitat.

The priority ecological corridors were Xiadu‐Hongxing, Changle‐Zhongqiao, Zhongqiao‐Shuangkeng, and Hongxing‐Shuangkeng, and the establishment of these corridors could play an important role in reducing the risk of population decline by increasing genetic selection pressure and promoting gene exchange (Alshwairikh et al. 2021; Li et al. 2023). However, these potential ecological corridors currently have a high proportion of residential areas and farmland. Due to the fear of alligators, alligators that disperse into human landscapes will likely be captured and returned to the nature reserves or blocked by alligator‐proof walls, resulting in the slow dispersal of alligators through these ecological corridors. Therefore, it will be difficult and complex to construct ecological corridors to connect the different nature reserves, and the effect of these ecological corridors on the alligator population will require long‐term observation and management (Sun et al. 2022).

Currently, most of the Chinese alligator conservation efforts are planned and managed by the Administrative Bureau of the Anhui Chinese Alligator National Nature Reserve. In addition to improving management within the reserve, the Administrative Bureau plans to add more long‐distance rivers and riverbanks in the reserves in the future to improve the connectivity of the habitat of the Chinese alligator in southern Anhui. Additionally, they intend to increase publicity on the harmlessness of the Chinese alligator and improve the compensation system for residents to reduce the resistance to the spread of Chinese alligators and increase the possibility of human‐crocodile coexistence in the same place.

Restoration of the Chinese alligator habitat depends on the construction of ecological corridors, which can be gradually achieved through the following methods. (1) Establishment of stepping‐stone habitats. The establishment of stepping‐stone habitats is a feasible method for ecological corridor construction and the restoration of habitat integrity (Ashrafzadeh et al. 2020), which can be used to gradually build short ecological corridors between the nature reserves. (2) Humans–alligators coexist ecological corridor design. Currently, most of the nature reserves of the Chinese alligator are close to agricultural areas. Based on the theory of fear (Smith et al. 2017), the residential areas around the reserve seriously limit the dispersal of Chinese alligators. Ecological corridors designed for the landscapes where both humans and alligators coexist, such as ecological greenways at the edge of urban and rural areas (Carlier and Moran 2019), could avoid the cost of large‐scale relocation of residents (Neelakantan et al. 2019). Moreover, to improve the dispersal of the Chinese alligator, crossing channels should be constructed to facilitate animal crossings, particularly when the ecological corridors intersect with highways or railways (Iannella et al. 2024).

In conclusion, based on the analysis of the niche model, the integrity of the Chinese alligator habitat was low, and the artificial landscape decreased the suitability of the habitat and limited the dispersal of the Chinese alligator. Construction of ecological corridors is likely to be difficult due to human activity and inappropriate landscapes. Various factors should be considered for ecological corridor construction between the reserves, especially coordinating human interests and the conservation of the Chinese alligator, and the integrity of the habitat could be restored by effective methods, such as building stepping‐stone habitats or human–alligator coexistence ecological corridors.

## Author Contributions

**Ke Sun:** formal analysis (equal), methodology (equal), software (equal), writing – original draft (equal). **Meng Li:** investigation (equal), writing – review and editing (equal). **Ziyi Wang:** investigation (equal), writing – review and editing (equal). **Siqing Sun:** resources (equal). **Jiayue Yang:** investigation (equal). **Xiaobing Wu:** conceptualization (equal), funding acquisition (equal). **Tao Pan:** conceptualization (equal), data curation (equal), funding acquisition (equal).

## Conflicts of Interest

The authors declare no conflicts of interest.

## Supporting information


Figure S1.

## Data Availability

Data was uploaded to figshare Dataset. DOI: https://doi.org/10.6084/m9.figshare.26928118.v2.
