# Supplementary figures and images for: Habitat Integrity Challenges for the Chinese Alligator Amid Land Occupation by Human: Pathways for Protection
Source: Ecol Evol. 2025 Mar 10;15(3):e71113. doi: 10.1002/ece3.71113 (PMC11893109; doi:10.1002/ece3.71113)

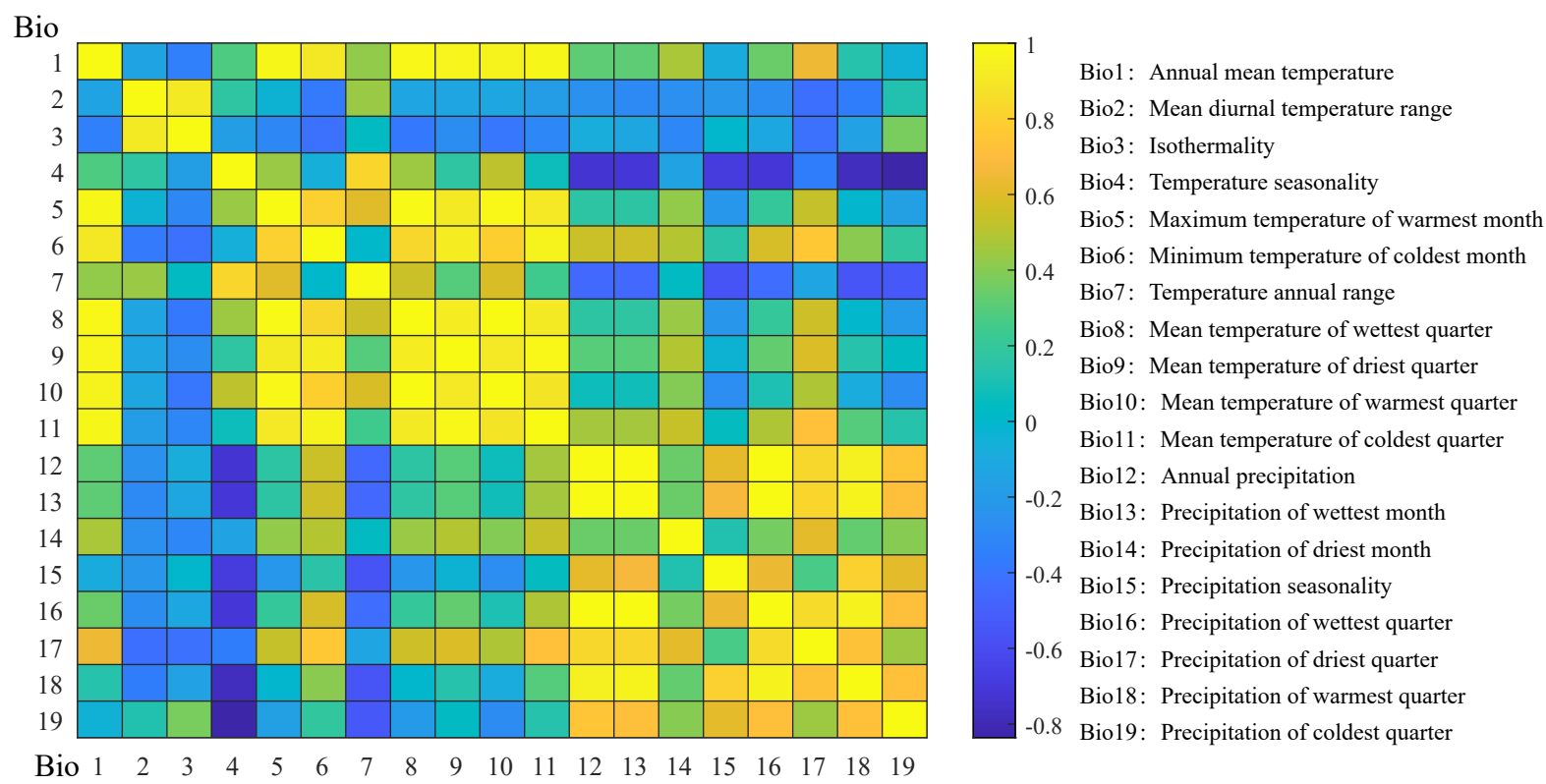

Supplement figure 1 Pearson correlation coefficients between the climate variables

Supplement: Supplementary file 1 — Figure S1. [file ECE3-15-e71113-s001.pdf]
